# Specific Quintuple: An On-Field Test to Predict Triple Jump Performance with High Accuracy

**DOI:** 10.5114/jhk/205292

**Published:** 2025-09-23

**Authors:** Claudio Mazzaufo, Mattia Beretta, Andrea Matarazzo, Gaspare Pavei, Stefano Serranò, Antonio La Torre, Gennaro Boccia

**Affiliations:** 1 Italian Athletic Federation (FIDAL).; 2 Department of Biotechnological and Applied Clinical Sciences, University of L’Aquila, L’Aquila, Italy.; 3 Department of Pathophysiology and Transplantation, University of Milan, Milan, Italy.; 4 Department of Biomedical Sciences for Health, University of Milan, Milan, Italy.; 5 Department of Clinical and Biological Sciences, University of Turin, Turin, Italy.

**Keywords:** track and field, jumping, performance prediction, testing

## Abstract

The triple jump is a demanding discipline in athletics, requiring physical capacity to cope with vertical ground reaction force peaks 8–15 times body weight when executed with a full run-up. This study investigated the potential of a specific drill executed with a shortened run up, the "specific quintuple", as a predictor of triple jump performance. A sample of 68 expert athletes participated in a total of 218 testing sessions, with measurements taken during both practice sessions and competitions. Linear hierarchical regression analysis revealed a strong correlation between performance of the specific quintuple drill and the triple jump competition performance (R2 = 0.96). This correlation remained significant across different age categories and sexes. The specific quintuple drill offers a safer and more gradual approach to skill acquisition, potentially mitigating the risk of overloading and injury, particularly among youth athletes. The study provides practical insights for coaches and athletes, offering regression equations for predicting triple jump performance based on specific quintuple performance. Overall, the specific quintuple drill emerges as a valuable tool for assessing and improving triple jump performance while prioritizing athletes’ safety.

## Introduction

The triple jump is an athletics event that consists of a run-up, a take-off, two successive jumps, the first of which is made with the same take-off leg, and a landing in the sand (Eissa, 2014; Miladinov and Bonov, 2004). The three jumps are referred to as the hop, the step, and the jump. During the second phase, vertical ground reaction forces can peak at 12–15 times body weight (Perttunen et al., 2000; Ramey and Williams, 1985). The triple jump is the discipline with the highest vertical force peaks among all athletics events. Indeed, in distance running 2–3 times body weight is reached (Cavanagh, 1987; Nilsson and Thorstensson, 1989; Pavei et al., 2017), in sprinting 4 times body weight (Plamondon and Roy, 1984; Rabita et al., 2015; Slawinski et al., 2017), and in the long jump 6–7 times body weight (Bosco, 1976; Ramey, 1970; Seyfarth et al., 1999). Such extreme loading makes the triple jump potentially hazardous for athletes who lack sufficient physical preparation or sound technical execution (Cao and Wang, 2023; Loturco et al., 2023). Athletes must develop the capacity to tolerate the mechanical stress associated with the event to minimize injury (Allado et al., 2021; Hay, 1992).

Even when athletes possess adequate technical and physical competencies, the high velocity of the run-up further increases both the difficulty and injury potential. Athletes reach the take-off with speeds exceeding 10 m/s (top male athletes) and 9 m/s (top female athletes), and produce parabolas of lengths exceeding 6 m for men and 5 m for women (Hommel, 2009; Hussain and Almujamay, 2022). For these reasons, the young athlete should be initiated into the event by following the proper didactic progressions: leaps and jumps should be proposed in various forms (alternating/successive/mixed). These drills should initially be performed on soft surfaces (lawn) with appropriate footwear to minimize traumatic events. Only with careful technique monitoring should the training volume of specific drills be progressively increased.

Practitioners’ experience has shown that youth athletes have to jump with relatively reduced run-ups in competitions. Unlike in other jumping events, few drills with full run-ups should be performed during practice so as not to stress the youth athlete further. For this reason, since the mid-1990s in Italy, technical specialists have proposed a specific technical drill called the “specific quintuple” with reduced run-ups. The specific quintuple is a succession of five connected mixed jumps (one classical triple and two alternating jumps). Based on the preferred right (R) or left (L) take-off leg, the sequence is: R-R-L-R-L, with landing in the sand, or L-L-R-L-R, with landing in the sand. The specific quintuple can be performed with a standing start with parallel feet or with a 2–4 step run-up during the learning phase for correct technical execution. During specific and competitive training periods the specific quintuple can be performed with a 6–8 (up to a maximum of 10–12) step run-up, in place of a full run-up. The main benefits of this drill include a gradual speed buildup toward the take-off, reduced flight distances during the first phase, and controlled transitions between each phase. Importantly, it also emphasizes maintaining velocity through the final phase (jump), performed with the same leg that completes the full triple jump. Due to its reduced mechanical demands, the specific quintuple is considered less traumatic than traditional full run-up jumping.

In the present study, we aimed to validate the specific quintuple as a test capable of predicting competitive triple jump performance in a large sample of high-level athletes. For this purpose, we aimed to corroborate the hypothesis that the specific quintuple could be a drill that might replace a full run-up during technical sessions of triple jumping training and, consequently, reduce its intrinsic traumatic nature and risk of injury.

## Methods

### Participants

Sixty-eight expert athletes, including 41 men and 27 women, participated in this study (mean age of 20.5 ± 4.5 years). As each athlete completed the protocol on multiple occasions (up to four times per athlete), a total of 218 testing sessions were performed. Each testing session included execution of both the specific quintuple drill and a competitive triple jump simulation (Table 1). All participants—categorized as under-18, under-20, under-23, or senior—had a personal best of at least 11 m for women and 13 m for men. In the U18 category, athletes completed 35 tests (28 tests were completed by male and 7 by female athletes), in the U20 category, 49 tests were performed (28 tests by male and 21 by female athletes), U23 athletes carried out 98 tests (59 tests were performed by male and 39 by female athletes), while seniors completed 36 tests (17 tests were completed by male and 19 by female athletes). The study was approved by the Institutional Ethics Committee of the University of Turin, Turin, Italy (protocol code: 510190; approval date: 17 November 2020).

**Table 1:** Descriptive statistics of the triple and quintuple jump performances.

	Triple jump performance (m)		Specific quintuple performance (m)	
**Age category**	**Males**	**Females**	**Males**	**Females**
U18	13.90 ± 0.48	11.67 ± 0.37	19.56 ± 1.05	16.30 ± 0.47
U20	14.49 ± 0.45	11.93 ± 0.67	20.52 ± 0.65	17.17 ± 0.94
U23	14.75 ± 0.78	12.24 ± 0.73	21.08 ± 1.09	17.63 ± 1.15
Senior	16.05 ± 1.09	13.92 ± 0.48	23.04 ± 1.70	18.30 ± 1.52

As shown in Table 1, participants were high-level athletes with experience in major international competitions, including World, European, and U23 Championships. Their achievements included multiple podium finishes, such as gold and silver medals at European events, two Diamond League victories, and medals at the Mediterranean Games.

### Design and Procedures

**Figure 1:**
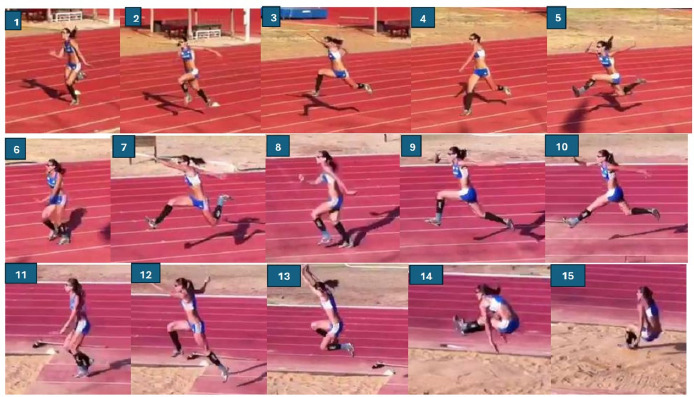
Photographic sequence of a female athlete performing the specific quintuple. The key phases are the following: 1) first take-off with the right leg, 4) second take-off with the right leg; 6) third take-off with the left leg; 8) fourth take-off with the right leg; 11) fifth take-off with the left leg; 15) landing in sand.

The specific quintuple test was conducted during technical training sessions held between eight and fifteen days prior to competition. Figure 1 presents a photographic sequence of a female athlete performing the specific quintuple. The distance covered during each specific quintuple was measured using a metric wheel. Testing sessions took place at various training facilities across Italy, including 53 official competition venues. All participating athletes were already familiar with the drill, as it was a regular part of their technical training.

Each athlete, wearing spiked shoes, was asked to perform two attempts of the specific quintuple (R-R-L-R-L-landing or L-L-R-L-R- landing, depending on their preferred take-off leg) with a 10-step run-up initiated from a standing start. The protocol for correct test execution during training was strictly followed, and all athletes received clear and consistent instructions.

In particular, we specified the meaning of a “standing start”: the athlete had to begin from a static position without any preparatory motion prior to the first stride, except for a natural loading movement used to initiate the run-up. Given the even number of steps, athletes began with the same foot they would later use for the take-off. The required movement sequence involved a triple jump followed by an additional alternating step and a final landing in the sand—totalling a hop, three steps, and a jump.

As per international regulations, the triple jump consists of a hop (landing on the same foot used for the take-off), a step (landing on the opposite foot), and a jump (concluding with a landing in the sand). In the specific quintuple, athletes were instructed to perform a hop, three bounding steps, and a jump, using the same take-off leg as in competition.

In competition, triple jump performance was measured using World Athletics-approved optical readers. Athletes selected their own run-up lengths—typically 14–16 steps for women and 16–18 steps for men. To measure the actual take-off point during training, high-speed video recordings (240 Hz) were captured using a tripod-mounted camera placed 2 m to the side and perpendicular to the take-off board. The take-off area was calibrated using cones and measuring tape placed at known distances. All recordings were analyzed using Kinovea software (version 0.9.3). During competitions, performance distances were obtained using Optojump technology (Microgate).

### Statistical Analysis

Linear hierarchical regression analyses were performed adopting the performance of the quintuple jump, the age category (U18, U20, U23, and Senior), and sex (males and females) as independent variables, and triple jump performance in official competition as the dependent variable. Since individual athletes performed the tests more than once, we considered the random intercept over athletes. Statistical significance of fixed effects was determined using type III Wald F tests, with Kenward-Roger degrees of freedom and the ANOVA function from the R’s car package (ver. 3.0.3) as follows:

**Triple jump in competition ~ quintuple jump × age category × sex + (1|athlete**)

After running the model, the residuals were checked for normality using the Shapiro-Wilk test. When the assumption of normality was violated, the residual outliers were removed using the Cook’s distance method (applying a distance of 3 standard deviations).

## Results

Triple jump performance was strongly predicted by specific quintuple performance (F = 123.07, *p* < 1×10^−16^). There was no interaction among specific quintuple performance × sex × age category (*p* = 0.528). The age category did not significantly affect triple jump performance either independently (*p* = 0.148) or in the interaction with specific quintuple performance (*p* = 0.095). Sex also did not influence the triple jump performance independently (*p* = 0.063) nor in the interaction with the age category (*p* = 0.602).

**Figure 2:**
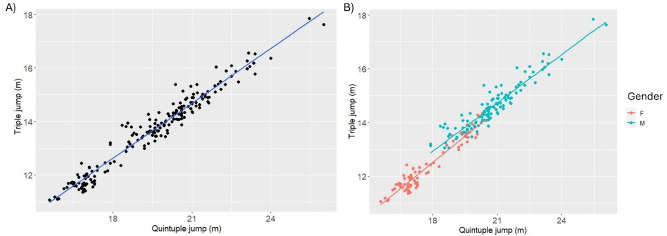
Scatter plot and linear regressions (modelled with the linear hierarchical model) between the specific quintuple test and triple jump performance in: A) the whole sample of athletes, and B) the sample of athletes grouped by sex. Each dot corresponds to a single test session. Each athlete performed multiple tests and competitions.

When including the whole sample of athletes, triple jump performance could be predicted by specific quintuple results using the following formula (Figure 2a):

**Triple jump in competition = (specific quintuple jump × 0.683) + 0.29 m; R2 = 0.96**

When grouping the sample of athletes by sex, triple jump performance could be predicted by specific quintuple results using the following formulae (Figure 2b):

Males: *Triple jump in competition = (specific quintuple jump × 0.591) + 2.28 m; R^2^ = 0.89*

Females: *Triple jump in competition = (specific quintuple jump × 0.654) + 0.71 m; R^2^ = 0.93*

**Figure 3:**
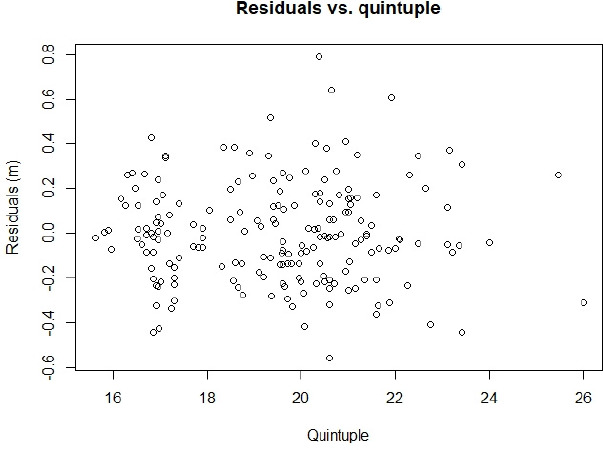
Residuals of the regression between the specific quintuple test and triple jump performance. The standard deviation of the residuals was ±0.30 m.

The residuals, reported in Figure 3, had a standard deviation of 0.30 m.

## Discussion

In the present study, a large sample of high-level national triple jumpers performed a specific on-field test several days prior to competition, on multiple occasions. We found that the test—referred to as the specific quintuple, which consists of five mixed jumps performed with a short run-up—was highly correlated with actual competition performance (R^2^ = 0.96).

These findings highlight the potential of the specific quintuple drill not only as a reliable predictor of triple jump performance, but also as a practical tool for reducing the injury risk associated with full run-up jumps. The inclusion of athletes from different age categories and of both sexes strengthens the generalizability of the results, offering robust support for the effectiveness of the specific quintuple as a performance assessment tool for the triple jump event.

Field tests are generally believed to correlate with actual triple jump performance in expert athletes (Moura et al., 2023). The standing quintuple jump (performed without a run-up) has previously been shown to have only a weak association with triple jump performance (r = 0.34) in a sample of university-level track and field athletes (Aoki et al., 2015). However, the present study validates a new specific test for triple jump prediction of competitions’ performance with high accuracy (R^2^ = 0.96). The strong correlation observed between performance in the specific quintuple drill and triple jump competition performance highlights the validity of the drill as a predictive tool. These findings also align with anecdotal observations commonly shared among Italian coaches who have long estimated that approximately 70% of the distance achieved in the specific quintuple corresponds to official triple jump performance (unpublished data). Importantly, a strong correlation was observed in this study despite the specific quintuple being executed with a shorter run-up (10 steps) compared to the longer run-up used in competition (14–18 steps). Athletes who performed well in the specific quintuple consistently achieved superior results in competition, suggesting that proficiency in the technical and physical demands of the drill translates effectively to competitive success.

Furthermore, the regression equations derived from this study offer practical value for coaches and athletes, enabling the prediction of triple jump performance based on results from the specific quintuple test. These equations provide a quantitative framework for evaluating an athlete’s readiness and potential, supporting the design of targeted training and development strategies tailored to individual needs. Notably, the standard deviation of the residuals was 30 cm, indicating that in approximately 68% of cases, triple jump performance could be predicted to within ±0.30 m using the specific quintuple test. This level of accuracy enables coaches to make more informed decisions regarding an athlete’s preparedness and to implement appropriate interventions to optimize their performance in upcoming competitions.

Importantly, the specific quintuple drill emerges as a promising alternative to full run-up triple jumps, particularly for youth athletes and those at greater risk of injury. By replicating the key technical components of the triple jump in a controlled and potentially less traumatic format, the specific quintuple offers a safer and more progressive pathway for skill acquisition and performance development. Its focus on gradual speed build-up, shorter flight phases, and controlled take-offs may help reduce the risk of overexertion and injury—especially in athletes with limited experience or insufficient physical conditioning. Moreover, incorporating the specific quintuple into training may encourage better distribution between the jump phases (Moura et al., 2023, 2024) without exposing the athlete to the high mechanical load associated with full run-up triple jumps. This makes it a valuable tool for injury prevention, technical refinement, and long-term athletic development. The present study focused exclusively on high-level athletes, meaning the generalizability of the findings to other populations, such as recreational or novice athletes, remains uncertain (Rodriguez-Gomez et al., 2024). Furthermore, while the specific quintuple drill appears to offer potential benefits to injury prevention and technical development, its long-term effectiveness and its comparative impact relative to traditional training methods require further investigation through longitudinal and controlled studies.

## Conclusions

In conclusion, the present study provides strong evidence supporting the validity and practical utility of the specific quintuple test as an effective tool for assessing the preparedness and projected competition performance of elite-level triple jumpers. The high correlation between test results and actual competitive outcomes indicates that this on-field assessment can serve as a valuable resource for coaches and sports scientists in evaluating athletes’ readiness. By closely replicating the physical and technical demands of the triple jump, the specific quintuple test offers an accurate reflection of an athlete’s capabilities, thereby enabling more informed decisions regarding the training design, competition planning, and performance optimization.
